# Levels of mannose-binding lectin (MBL) associates with sepsis-related in-hospital mortality in women

**DOI:** 10.1186/s12950-020-00257-1

**Published:** 2020-08-12

**Authors:** Sofie Jacobson, Peter Larsson, Anna-Maja Åberg, Göran Johansson, Ola Winsö, Stefan Söderberg

**Affiliations:** 1 Department of Surgical and Perioperative Sciences, Anesthesiology and Intensive Care Medicine, Umeå University, SE-901 87 Umeå, Sweden; 2 Department of Public Health and Clinical Medicine, Medicine, Umeå University, SE-901 87 Umeå, Sweden

**Keywords:** Sepsis, Mannose-binding lectin, Sex, Case-referent study

## Abstract

**Background:**

Mannose-binding lectin (MBL) mediates the innate immune response either through direct opsonisation of microorganisms or through activation of the complement system. There are conflicting data whether MBL deficiency leads to increased susceptibility to infections or not. The aim of this study was to determine if low levels of mannose-binding lectin (MBL) predict sepsis development, sepsis severity and outcome from severe sepsis or septic shock.

**Method:**

Patients aged 18 years or more with documented sepsis within 24 h after admission to the intensive care unit were included if they had participated in a health survey and donated blood samples prior to the sepsis event. A subset of these patients had stored plasma also from the acute phase. Two matched referents free of known sepsis were selected for each case. Plasma levels MBL were determined in stored samples from health surveys (baseline) and from ICU admission (acute phase). The association between MBL and sepsis, sepsis severity and in-hospital mortality were determined with 1300 ng/mL as cut-off for low levels.

**Results:**

We identified 148 patients (61.5% women) with a first-time sepsis event 6.5 years (median with IQR 7.7) after participation in a health survey, of which 122 also had samples from the acute septic phase. Both high MBL levels in the acute phase (odds ratio [95% confidence interval]) (2.84 [1.20–6.26]), and an increase in MBL levels from baseline to the acute phase (3.76 [1.21–11.72]) were associated with increased risk for in-hospital death in women, but not in men (0.47 [0.11–2.06]). Baseline MBL levels did not predict future sepsis, sepsis severity or in-hospital mortality.

**Conclusions:**

An increase from baseline to the acute phase as well as high levels in the acute phase associated with an unfavourable outcome in women.

## Background

Severe sepsis is a life-threatening syndrome where little is known about factors determining susceptibility for developing the syndrome and severity of the syndrome once developed. Potentially, biomarkers could be used for identifying those at risk for severe sepsis needing aggressive treatment, which is subject for intensive studies [1–4] . With advances in recombinant techniques, targeted substitution therapies are forthcoming and one of the challenges will be to define which patients will benefit from such therapies [5–7].

Mannose-binding lectin (MBL) is a serine protease belonging to the collectin family and is believed to be an important factor in the innate immune system, the first line host defence. With its pattern-recognizing ability, MBL binds to the surface of a wide range of microorganisms, although not all, thus functioning either as a direct opsonin, or through activation of the complement system, thereby enhancing phagocytosis of microorganisms by macrophages and neutrophils [8].

There are several known mutations in the structural *MBL2*-gene and its promoter regions located at the long arm of chromosome 10, resulting in a large number of haplotypes. This genetic polymorphism is associated with different levels of MBL expression and activity [9–13].

There are several reports indicating that genotypes associated with low levels of MBL may predispose to certain forms of infection or impaired immune response, particularly in new-borns, but also in adults [14–17]. Other reports indicate that low levels of MBL may augment the humoral immune system [18–23]. However, there are considerable overlaps in MBL concentrations between different genotypes, and inter-individual variability among individuals with identical genotypes is significant [10–13, 24, 25]. Different definitions and cut-off values have been used to define MBL deficiency [17, 26–29] and genotypes associated with low MBL production are common, as high as 25–30% in certain populations [25, 29].

Several studies on the association of MBL genetic polymorphism and/or MBL plasma levels with severe infections, sepsis and septic shock, have shown an increased risk of sepsis development and unfavourable outcome in MBL deficient patients [27, 28, 30, 31]. However, there are conflicting results [26, 32–34].

In this nested case-referent study we hypothesized that low MBL levels associate with increased risk of future sepsis, and its severity, and in contrast, that high levels associates with decreased risk of sepsis and sepsis related mortality. Furthermore, that the MBL-associated risk is similar in men and women.

## Material and methods

Design and methods have been previously reported [35]. Shortly, cases were identified retrospectively within health survey cohorts and biomarkers were analyzed in blood samples collected at the health surveys (baseline) and when available, in blood samples collected at admission to the intensive care unit (acute phase).

A total of 797 patients were admitted with a diagnosis of sepsis at the Intensive Care Unit, Umeå University Hospital, Sweden, between 1 March 1988 and 31 October 2008.

The diagnosis of sepsis and the severity of sepsis were confirmed retrospectively by reviewing hospital records, including results from biochemical, microbiological, and radiological examinations.

Of the 797 patients, 148 had prior to the septic event participated in one of four population-based health studies in Northern Sweden: the Västerbotten Intervention Program (VIP), the Northern Sweden MONItoring Of trends and Determinants in CArdivascular Disease (MONICA) survey, the Mammary Screening Program (MSP), and the Northern Sweden Maternity Cohort (NSMC). The contribution of cases from each survey was 80 (VIP), 4 (MONICA), 42 (MSP), and 22 (NSMC). The Northern Sweden Health and Disease Study (NSHDS) which includes the three former studies, and the Northern Sweden Maternity Cohort (NSMC) are described in detail in our previous report [35].

Patients aged 18 years or older were included if they had a diagnosis of sepsis within 24 h after admittance to the ICU. Only the first event was included for patients with multiple admissions due to sepsis. The Third International Consensus Definitions for Sepsis and Septic Shock (Sepsis-3) were used [36]. Acute Physiology, Age, Chronic Health Evaluation II score (APACHE II) was calculated for assessment of severity of illness at admission [37]. Sequential Organ Failure Assessment Score (SOFA) was calculated as a marker for organ dysfunction and disease severity [38].

Data on length of stay, mortality, referral patterns, and reasons for admission, co-morbidities, and sources of infection, primary infection sites and causative microorganisms were collected. Microbiological cultures acquired within 48 h before or after admission to the ICU were considered relevant. Pre-existing diseases were defined according to Knaus et al. [37].

For each case, two referents without any episode of sepsis and being alive at the date of the case admission to ICU were chosen and matched for age (± 2 years), gender, health survey, and time of blood sampling (± 30 days). Matching on smoking (y/n) was incomplete due to missing information, mainly in the MSP.

In addition, 122 out of 148 patients had also retrievable samples collected at ICU admission (the acute phase). Thus, 122 patients had samples from both the health survey examination (baseline) and from admission to the ICU (the acute phase).

The study protocol was approved by the Regional Ethical Review Board in Umeå and by the Swedish National Computer Data Inspection Board, and complies with the Declaration of Helsinki. All participants gave written informed consent for future use of data and blood samples.

### Chemical analyses

MBL in plasma was analysed in duplicates using a commercially available ELISA (MBL Oligomer ELISA Kit 029, BioPorto Diagnostics, Gentofte, Denmark) in accordance with the instructions from the manufacturer. The absorbance was read on a spectrophotometer (Labsystems Multiskan MS, Triad Scientific Inc., USA). The range of the assay was 0 to 4000 ng/mL. The distribution of MBL in healthy Danish blood donors analysed with the same assay was provided by the manufacturer. There was no significant difference in MBL distribution when comparing the Danish cohort with our study cohort (Supplementary Table 5 and 6).

### Statistical analyses

Data are presented as numerical values or percentages. Continuous data are presented as median with interquartile range. For comparisons, Fisher’s exact, Mann-Whitney U-tests or Wilcoxons Signed Rank test were used when appropriate. Spearman correlation test was used for correlation. Since cases and referents had the same follow-up time within strata in this nested and matched case-referent study, logistic regression analysis (rather than Cox regression) using the conditional maximum likelihood routine designed for matched analysis was used to estimate odds ratios with 95% confidence intervals (CI), and the influence of MBL on future sepsis was tested in a univariable model. Non-conditional logistic regression (only cases) analysis was used to calculate the risk for in-hospital death. Mannose-binding lectin was tested as a categorical variable with 1300 ng/ml as cut-off for low levels [27]. The accuracy of this cut-off was tested with Receiver Operator Characteristic (ROC) graphs with calculation of the area under the curve (AUC).

The change in MBL levels from baseline to the acute phase (the difference in MBL levels between base line and the acute phase) was also tested as a categorical variable. The cut-off was set at zero, with positive values representing an increase and negative values representing a decrease from baseline to the acute phase. The accuracy of this cut-off was also tested with ROC analysis. A *p*-value < 0.05 was considered significant, and all *p*-values reported are two-sided. SPSS ver. 24 was used for statistical analysis.

## Results

Sixty-one percent of both cases and referents were women (matched), and there was no difference in age between cases and referents (matched) but women were younger than men at baseline survey, 49.8 years and 53.2 years, respectively (*P* = 0.003). Cases had marginally higher BMI than referents (*P* = 0.04) but the prevalence of diabetes, hypertension, hypercholesterolemia and smoking did not differ (Table 1).

**Table 1:** Subject characteristics at baseline surveys

	*n* = cases/referents	Cases	95% CI	Referents	95% CI	*p*
Age years	148/296	51.1	49.0–53.2	51.1	49.6–52.6	*(matched)*
Female gender, %	91/182	61.5	53.6–69.4	61.5	55.9–67.1	*(matched)*
BMI, kg/m^2^	116/229	27.6	26.5–28.7	26.4	25.8–26.9	*0.04*
Reduced glucose tolerance, %	80/151	35.0	24.3–45.7	23.8	17.0–30.7	*0.16*
Daily smoker, % #	116/237	28.4	20.1–36.8	28.7	22.9–34.5	*(matched)*
Hypertension, %	83/158	55.4	44.5–66.4	43.0	35.2–50.8	*0.08*
Systolic BP, mmHg	83/158	135	130–139	132	129–135	*0.34*
Diastolic BP, mmHg	83/158	82	80–85	82	80–83	*0.74*
Cholesterol, mmol/L¤	83/155	5.7	5.4–5.9	6.0	5.8–6.2	*0.06*

Values reported are means or percentages % with 95% CI. Hypertension was defined as systolic BP > 140 mmHg and/or diastolic BP > 90 mmHg and/or antihypertensive treatment. Reduced glucose tolerance included any of IFG, IGT or DM. Referents were matched with cases based on age, sex and (if available) smoking status

*Abbreviations*: *DM* Diabetes mellitus, *IFG* Impaired fasting glucose, *IGT* Impaired glucose tolerance, *BP* Blood pressure

**Fig. 1:**
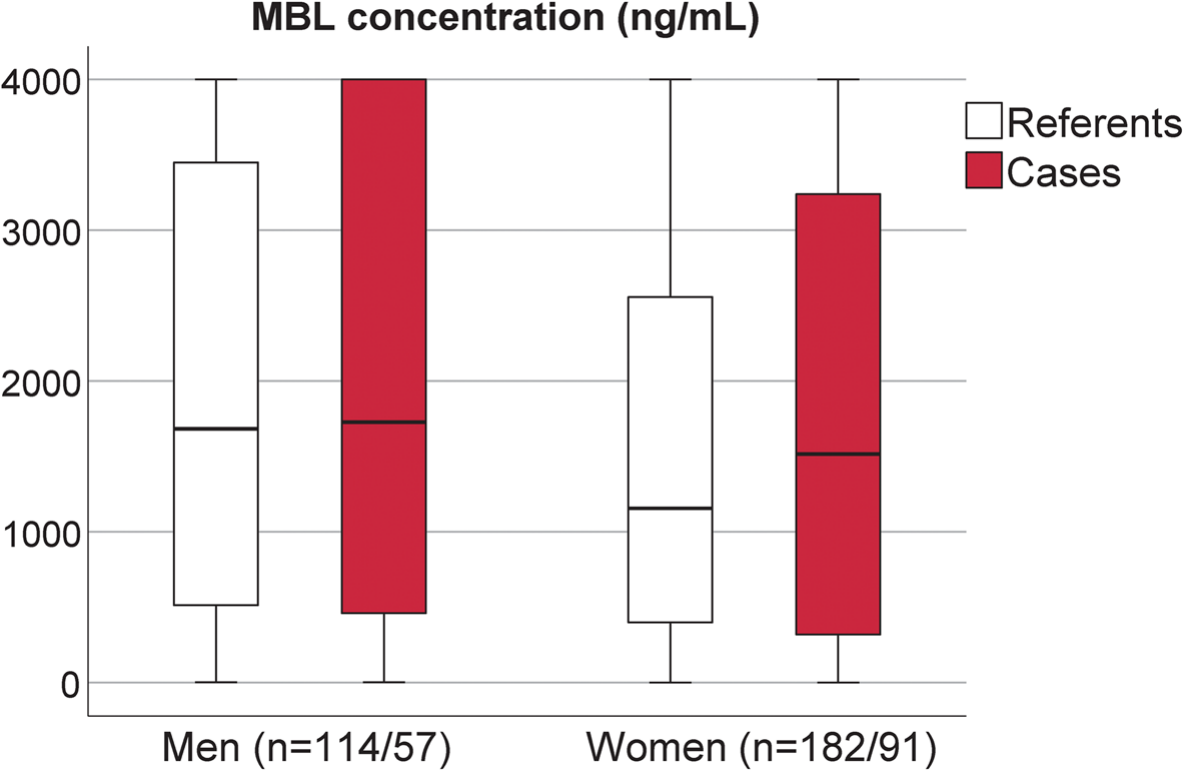
MBL concentrations (ng/mL) at baseline survey for men and women, cases and referents. Men are displayed to the left in the panel and women to the right. Empty boxes represent referents and filled boxes represent cases. Data are presented as median and interquartile range (IRQ)

Circulating MBL levels at baseline did not differ between cases and referents (*P* = 0.5) (Table 2). However, when comparing men and women in the whole cohort women had slightly lower levels at baseline than men (*P* = 0.04), but there was no difference between female cases and their referents (*P* = 0.5), or between male cases and their referents (*P* = 0.9) (Fig. 1). Further, the distribution of low and high MBL levels (cut-off 1300 ng/mL) did not differ between cases and referents or between men and women (Table 2).

**Table 2:** MBL concentration (ng/mL) at baseline survey for cases and referents

	Cases	Referents	Cases	Referents
	n	n	MBL (ng/mL)	MBL (ng/mL)
MBL conc. (ng/mL)	148	296	1646 (IQR 3095)	1401 (IQR 2305)
MBL < 1300 (ng/mL)	63 (42.6%)	140 (47.3%)	284 (IQR 541)	434 (IQR 576)
MBL > = 1300 (ng/mL)	85 (57.4%)	156 (52.7%)	2863 (IQR 2170)	2671 (IQR 2101)

Data are presented as numbers (%) and median with interquartile range (IQR)

Patient characteristics at ICU admission are shown in Table 3. Women were younger when developing sepsis (*P* = 0.04), and the period between the baseline survey and the sepsis event was 7.4 years (IQR 7.8) for men and 5.9 years (IQR 8.1) for women (*P* = 0.04). Of the events, 67% were classified as severe sepsis and 33% as the most severe form, the septic shock. Severity of sepsis, APACHE II- and SOFA score, length of stay, ICU- and in-hospital mortality did not differ between men and women. There were no differences in co-morbidities, sources of infection and infecting microorganisms between men and women except for infections with gram negative rods which was slightly more frequent in men (*P* = 0.04).

**Table 3:** Patient characteristics at ICU admission

	All patients (*n* = 148)	Men (*n* = 57)	Women (*n* = 91)
Age years	60.7 (IQR 18.3)	63.4 (IQR 16.5)	57.2 (IQR 18.0)
Years between survey and sepsis	6.5 (IQR 7.7)	7.4 (IQR 7.8)	5.9 (IQR 8.1) *
BMI (*n* = 116, 52/64)	26.7 (IQR 6.0)	26.0 (IQR 6.2)	26.9 (IQR 7.0)
Disease scores			
APACHE II Score	18.0 (IQR 9)	17.0 (IQR 10)	18.0 (IQR 9)
SOFA score	7.0 (IQR 5)	7.0 (IQR 5)	7.0 (IQR 6)
Disease severity, n (%)			
Severe sepsis	99 (66.9%)	42 (74%)	57 (63%)
Septic shock	49 (33.1%)	15 (26%)	34 (37%)
ICU mortality	27 (18.2%)	10 (17.5%)	17 (18.7%)
Hospital mortality	32 (21.6%)	13 (22.8%)	19 (20.9%)
Hospital Length of stay	17.5 (IQR 24.7)	15 (IQR 25.5)	18 (IQR 27)
Co-morbidities, n (%)			
COPD	4 (2.7%)	1 (1.8%)	3 (3.3%)
Congestive heart failure	5 (3.4%)	3 (5.3%)	2 (2.2%)
Chronic renal insufficiency	4 (2.7%)	2 (3.5%)	2 (2.2%)
Chronic liver disease	0 (0%)	0 (0%)	0 (0%)
Diabetes, n (%)			
Insulin treatment	11 (7.4%)	4 (7.0%)	7 (7.7%)
Other treatments	8 (5.4%)	4 (7.0%)	4 (4.4%)
Cancer, n (%)			
Hematological	9 (6.1%)	4 (7.0%)	5 (5.5%)
Localized	20 (13.5%)	9 (15.8%)	11 (12.1%)
Metastatic	11 (7.4%)	5 (8.8%)	6 (6.6%)
Immunosuppressants, n (%)			
Chronic steroids	8 (5.4%)	3 (5.3%)	5 (5.5%)
Chemotherapy	13 (8.8%)	7 (12.3%)	6 (6.6%)
Other immunosuppression	12 (8.1%)	4 (7.0%)	8 (8.8%)
Primary infection site, n (%)			
Pneumonia	24 (16.2%)	11 (19%)	13 (14%)
Abdominopelvic	50 (33.8%)	19 (33%)	31 (34%)
Urinary tract	20 (13.5%)	10 (18%)	10 (11%)
Other	52 (35.1%)	17 (30%)	35 (38%)
Unknown	5 (3.4%)	2 (3.5%)	3 (3.3%)
Infecting microorganism, n (%)			
Gram positive (cocci)	62 (14.0%)	23 (40.4%)	39 (42.9%)
Gram negative (rods)	40 (27%)	21 (36.8%)	19 (20.9%) *
Fungi	11 (7.4%)	3 (5.3%)	8 (8.8%)
Virus	9 (6.1%)	3 (5.3%)	6 (6.6%)
Negative cultures	33 (22.3%)	13 (22.8%)	20 (22.0%)

Data are presented as numbers (%) or median and interquartile range (IQR)

* *p* < 0.05 Mann-Whitney and Chi2. *Abbreviations*: *APACHE* Acute Physiology, Age and Chronic Health Evaluation, *SOFA* Sequential Organ Failure Assessment, *CI* Confidence interval, *IQR* Interquartile range, *COPD* Chronic obstructive pulmonary disease, *ICU* Intensive care unit

**Fig. 2:**
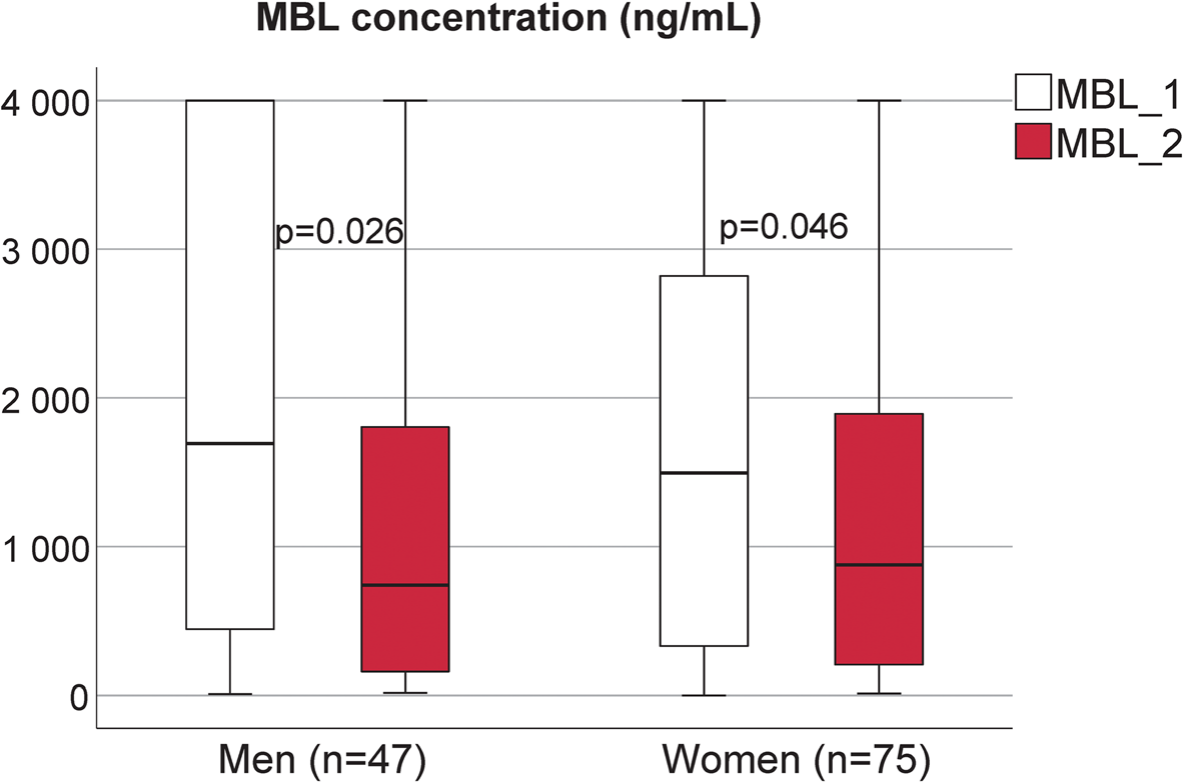
MBL concentrations (ng/mL) at baseline (MBL1) and in the acute phase (MBL2) for men and women. Men are displayed to the left in the panel and women to the right. Empty boxes represent baseline levels and filled boxes represent levels in the acute phase. Data are presented as median and interquartile range (IRQ). * *p* < 0.05 MBL2 vs. MBL1 using Wilcoxon Signed Rank test

MBL levels at ICU admission (samples available from 122 patients) did not differ between men and women (*P* = 0.7). However, MBL levels in the acute phase were significantly lower than baseline levels in both men and women (*P* = 0.03 and *P* < 0.05, respectively) (Fig. 2). Co-morbidities, sources of infection and infecting microorganisms did not differ in those with low MBL levels defined as levels below 1300 ng/mL compared to patients with MBLlevels above 1300 ng/mL (data not shown).

Circulating MBL levels at baseline did not correlate with age (*r* = − 0.02, *P* < 0.8), BMI (*r* = − 0.02, *P* < 0.9), fasting or post-load glucose levels, (*r* = 0.05, *P* = 0.6 and *r* = − 0.07, *P* = 0.5, respectively), or with systolic or diastolic blood pressures (*r* = − 0.07, *P* = 0.6 and *r* = − 0.15, *P* = 0.2, respectively). Similarly, MBL levels in the acute phase did not correlate with age (*r* = 0.09, *P* = 0.3), BMI (*r* = − 0.02, *P* = 0.9), APACHE II score (*r* = 0.10, *P* = 0.3), or SOFA score (*r* = 0.08, *P* = 0.4). Circulating levels of MBL in the acute phase did not correlate to MBL at baseline (*r* = 0.004, *P* = 1.0). Correlation analysis stratified by sex did not add any more information, neither at baseline nor in the acute phase (data not shown).

Low levels at baseline expressed as circulating MBL below 1300 ng/mL did not predict a future sepsis event (0.82 [0.55–1.23]), or increased severity; severe sepsis (0.94 [0.58–1.54]), septic shock (0.64 [0.32–1.27]), or hospital death (1.29 [0.54–3.08]). Similar point estimates were seen when stratified for sex. Several other cut-offs were also tested, but lower levels (than 1300 ng/mL) did not associate with future sepsis development, sepsis severity or hospital outcome (Supplementary Table 4).

**Fig. 3:**
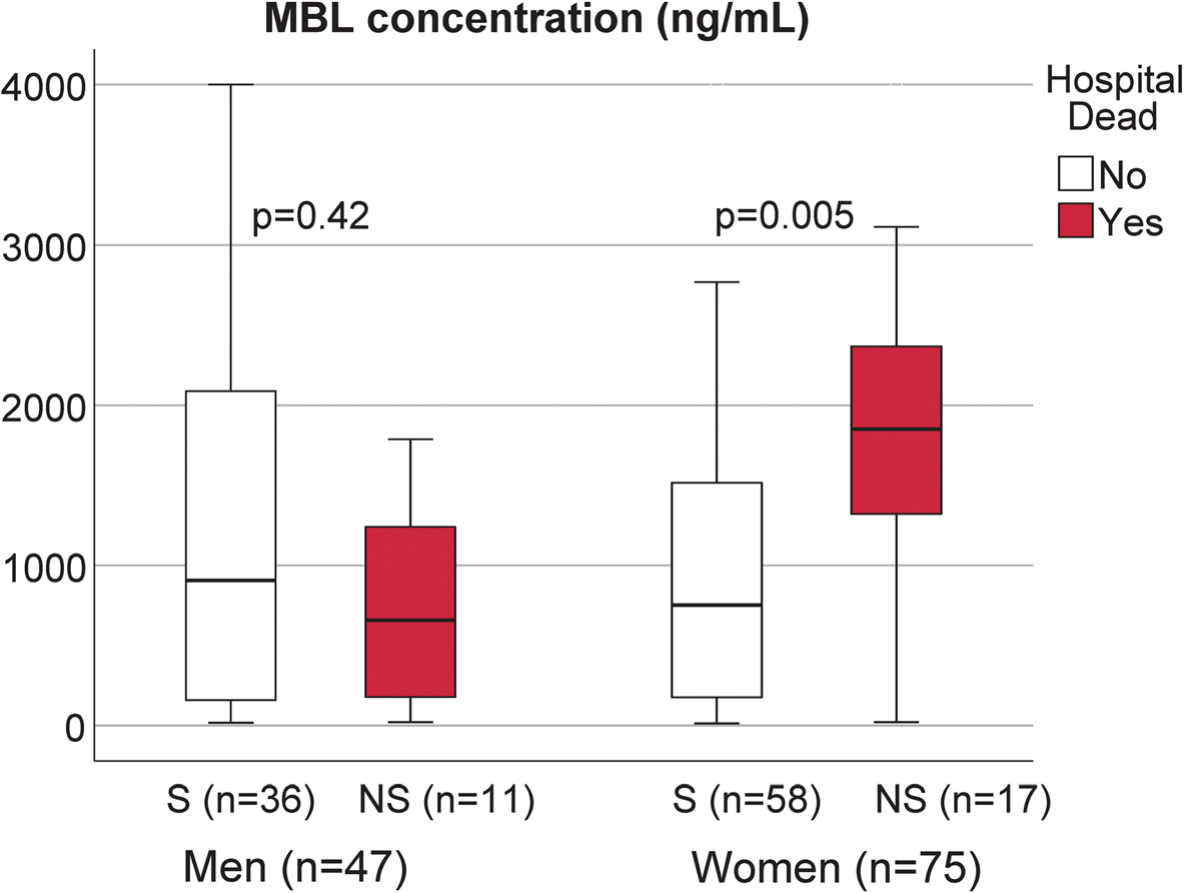
MBL concentrations (ng/mL) in the acute phase for men and women, survivors (S) and non-survivors (NS). Men are displayed to the left in the panel and women to the right. Empty boxes represent survivors and filled boxes represent non-survivors. Data are presented as median and interquartile range (IRQ). * *p* < 0.05 NS vs. S using Mann-Whitney U-test

Women who died had significantly higher levels in the acute phase than surviving women (*P* = 0.005), and they had also higher levels than men who died (*P* = 0.02) (Fig. 3).

**Fig. 4:**
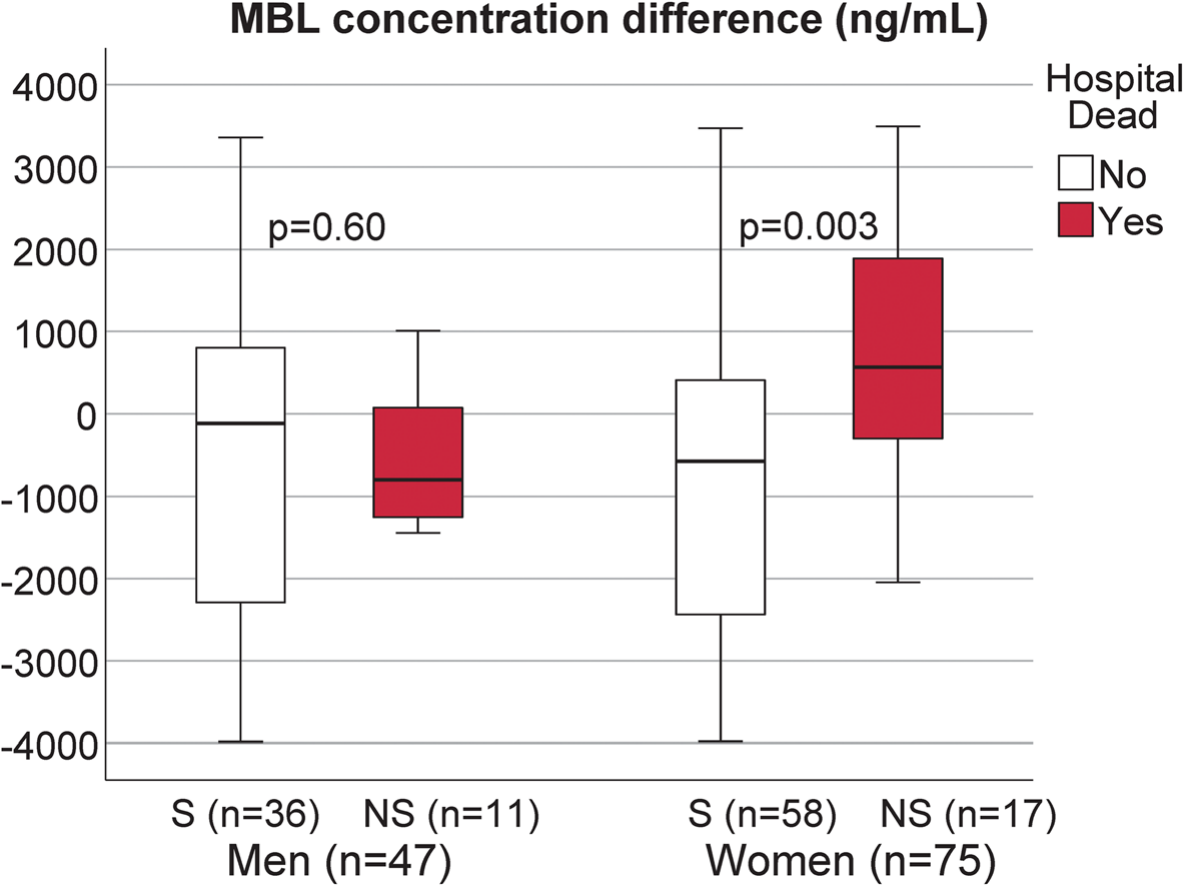
MBL (ng/mL). Differences between MBL levels in the acute phase and at baseline for survivors (S) and non-survivors (NS) displayed for men and women separately. Men are displayed to the left in the panel, and women to the right. Empty boxes represent survivors and filled boxes represent non-survivors. For each box a negative value represent a decrease and a positive value represents an increase, (MBL_acute phase - MBL_baseline) > 0 = increase from baseline to the acute phase, (MBL_acute phase - MBL_baseline) < 0 = decrease from baseline to the acute phase. Data are presented as median and interquartile range (IRQ). * *p* < 0.05 NS vs. S using Mann-Whitney U-test

Intra-individual MBL levels decreased significantly from baseline to the acute phase in women who survived (*P* = 0.002). Further, there was a significant difference in the change of MBL levels in surviving women compared to non-surviving women (*P* = 0.003). In men, the intra individual changes did not differ between survivors or non-survivors (*P* = 0.6) (Fig. 4).

The association between MBL levels in the acute phase and in-hospital death was analysed, with 1300 ng/mL as cut-off. The accuracy of the chosen cut-off (1300 ng/mL) was tested in a ROC analysis showing a diagnostic accuracy of 65.6% at 1319 ng/mL, with a sensitivity of 68.1% and specificity of 57.1% for the whole group. AUC was 0.60 (0.48–0.72, 95%CI), *p* = 0.044. For women, the ROC analysis showed a diagnostic accuracy of 73.3% at 1319 ng/mL, with a sensitivity of 72.4% and specificity of 76.5%. AUC was 0.73 (0.59–0.86, 95%CI), *P* = 0.0008. For men, no discrimination point was detected (Supplementary Figures 7, 8 and 9, respectively).

**Fig. 5:**
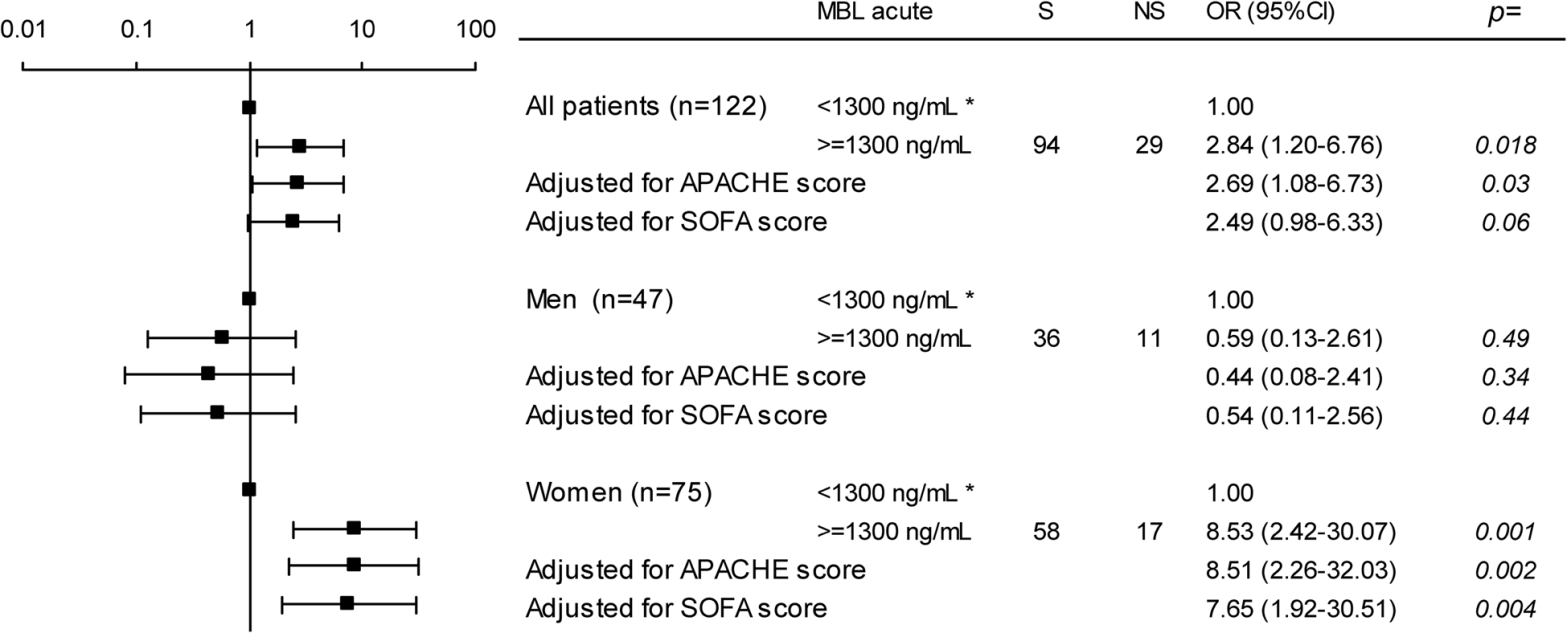
Logistic regression. MBL concentration in the acute phase < 1300* vs. > = 1300 ng/mL and the risk of in-hospital death. All patients are displayed in the upper part of the panel, men in the middle part of the panel and women in the lower part of the panel. First as univariate analysis and then APACHE II and SOFA scores are introduced separately. Data are presented as odds ratio (OR) and 95% confidence intervals. * denotes the indicator contrast with OR 1.00

**Fig. 6:**
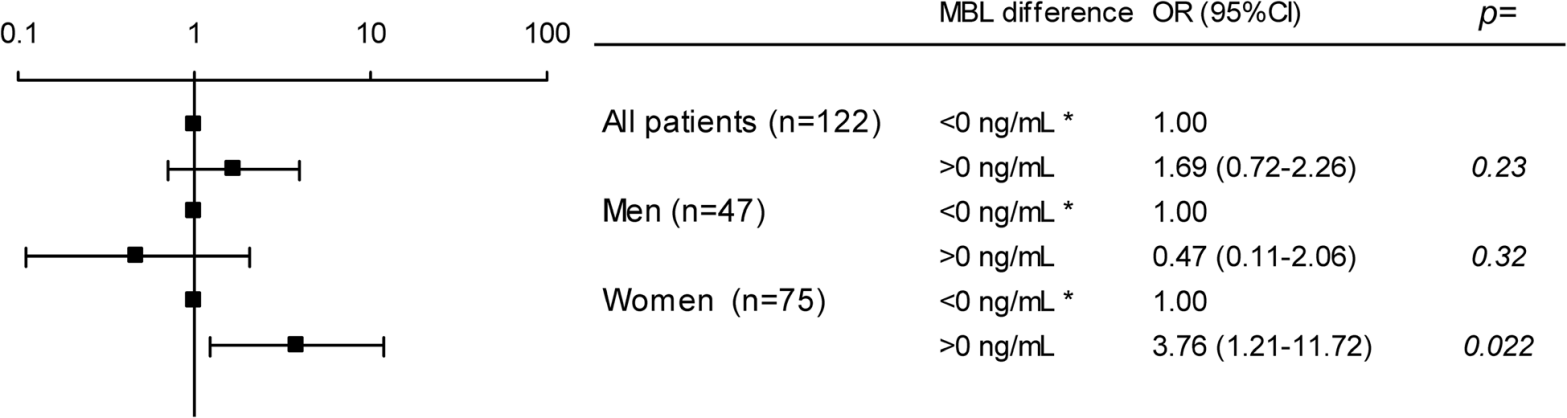
Univariate logistic regression. The difference in MBL levels between the acute phase and baseline (MBL_acute phase - MBL_baseline) and the risk of in-hospital death. All patients are displayed in the upper part of the panel, men in the middle part of the panel and women in the lower part of the panel. Data are presented as odds ratio (OR) and 95% confidence intervals. * denotes the indicator contrast with OR 1.00

High levels associated with in-hospital death (2.84 [1.20–6.76]). In the stratified analysis, the association remained in women (8.53 [2.42–30.07] but not in men (0.59 [0.13–2.61]). The association remained for women even after adjustment for APACHE II score and for SOFA score separately (Fig. 5). Furthermore, an increase from baseline to the acute phase associated with hospital death in women (3.76 [1.27–11.72]) but not in men (0.47 [0.11–2.06]) (Fig. 6). In a ROC analysis a diagnostic accuracy of 73.3% for the risk of in hospital death for women was found at an increase of 516 ng/mL from baseline to the acute phase, with a sensitivity of 75.9% and specificity of 64.7%. AUC was 0.74 (0.61–0.87, 95%CI), *p* = 0.0001 but no discriminating point could be seen in men, (Supplementary Figure 10 and 11, respectively).

## Discussion

We report that low levels of MBL in a pre-sepsis state did not associate with a future sepsis event. Further, in the acute phase of sepsis there were sex-related differences in MBL levels in relation to in-hospital mortality. Women who died in hospital had higher levels than surviving women. This association between MBL levels and outcome was not seen in men. Furthermore, an increase in MBL levels from baseline to the acute phase associated with hospital death in women but not in men. To our knowledge, the association between circulating MBL levels and sepsis outcome in women has not been previously reported. This finding indicates the presence of sex-related differences related to innate immunity. It has been reported that ‘resting’ MBL levels in women may be higher than those of men of similar age, though others have not made such observation [39, 40].

A second notable finding relates to changes in MBL levels in response to acute sepsis. Contrary to the expected, MBL levels decreased from baseline to the acute phase, especially in survivors, with a differential pattern in men and women.

The notion of MBL as an acute phase reactant stem from earlier findings. The promoter sequence of the MLB2 gene contains several consensus elements. As in other acute phase reactants, the transcription is enhanced by IL-6, dexamethasone and heat shock protein but inhibited by IL-1 [41, 42]. However, MBLs function as an acute phase reactant has been questioned since there is considerable heterogeneity in the acute response in different settings and the influence of genetic polymorphism is substantial. A slower and less obvious MBL response to infection or surgical trauma compared with other acute phase reactants, as C-reactive protein, and variable responses in sepsis have been reported [43–46]. In an Australian study, 41% of patients with pneumonia and blood stream infection had stable MBL levels through their hospital stay [44]. Further, they found that patients who were MBL deficient at study entry, failed to demonstrate a positive acute phase response into the normal range. This diverge from our results were 35% of patients with baseline values less than 500 ng/mL increased to 1500 ng/mL or more in the acute phase. Differences in patient selection and timing of blood sampling may account for these discrepancies.

Another main finding was that low pre-sepsis MBL levels were not identified as a risk for severe sepsis, septic shock or unfavourable outcome. This is contrary to some interpretations that low MBL levels or a state of MBL deficiency is associated with increased risk for infection and development of SIRS, sepsis, septic shock, and even sepsis related death [28, 30, 47]. Also, in this respect there are conflicting data. In a large population based study were 9245 individuals were genotyped and followed 8 to 24 years, no evidence for significant differences in infectious disease or mortality in MBL deficient individuals versus controls was found [32]. A study on intensive care patients could not find any difference in frequency of MBL2-polymorfism between patients and controls at baseline, and between patients classified as having sepsis or not [34].

Considering the biological function of MBL as a pathogen recognizing molecule that either directly or mediated via the associated serinproteases MAPS2 activate the complement pathway and enhance phagocytosis, it would not be surprising if a ready access of functional MBL are beneficial in case of an infection. However, if this leads to an exaggerated complement activation it could result in extensive tissue damage, detrimental for the host. Of note in our cohort of patients, none of the women with the highest MBL values at baseline died in hospital, while an increase or high levels in the acute phase did not prove to be beneficial, at least not for women. In theory, this could imply that an abundance of MBL allowing a rapid pathogen recognition and early neutralization prevents further, uncontrolled activation of other cascade systems with subsequent excessive inflammatory response and organ dysfunction. A delayed response with an increase of MBL when other components of the innate immune system already are set into action may impose additional, non-beneficial inflammatory responses. Partly supporting this notion are data indicating beneficial effects of low MBL levels in different settings [18–21, 23, 48–50]. Thus, MBL may have different effects in different situations and in different phases of acute illness. An alternative explanation for the finding that patients who died had higher MBL levels than survivors could relate to dysfunctional MBL with reduced ligand binding and opsonin function which could lead to reduced phagocytosis and reduced clearance from the circulation and higher free MBL levels. However, the assay used in this study is considered to predominantly detect oligomeric or “functional” MBL.

Most studies have not considered the possibility of sex-related differences and data are not presented stratified for sex. The value of MBL as a prognostic marker for out-come or patient selection for substitution or inhibitory therapy requires a deeper understanding of its action before implementation into clinical practice.

We were not able to find any association between MBL levels and degree of severity of acute sepsis, source of infection, infecting microorganism or other comorbidities. This was unexpected, since acute illness and co-morbidities might be expected to be accompanied by some degree of inflammation, also with corresponding MBL expression. A finding also unexpected in the light of reports that MBL deficiency is associated with recurrent respiratory infections and infections with gram-positive bacteria [16, 51–53]. However, the power to detect more subtle associations may have been restricted by the size of the study cohort.

Our results contradict findings from others that the presence of MBL variant alleles, and low MBL levels, associates with the development of sepsis, severe sepsis, and septic shock [28, 30]. There are also others who have not been able to show such distinct associations between severity of illness and MBL levels or genotype [26, 34, 44].

### Limitations in the study design

In this study, only circulating plasma levels of MBL were determined, which ideally should have been combined with genotypes and studies of MBL function. More than 80 polymorphic sites are known, not all of known clinical relevance and only seven haplotypes are commonly found and studied were three different structural variants, B, C and D and the promoter haplotypes HY, LY and LX have a dominant effect on circulating MBL levels [10, 25, 33]. However, an individual’s MBL levels cannot be determined from its genetic variant alone, since there are considerable inter-individual variations and other yet unknown factors probably influence circulating levels [12, 13, 25, 33]. Still, a future study of sex-related MBL responses would ideally include genotyping. Further, due to lack of resources we were not able to specifically analyse MBL function, as with analysis of C4b deposit or MBL associated serine protease 2 (MASP-2).

The sample size was determined by access to pre-illness biomaterial, which affected the patient selection and limited the number of observations. For this reason, there is a majority of women of slightly younger age than the men. Patients do not entirely represent all patients consecutively admitted to the ICU, though their characteristics are representative for patients with sepsis at our ICU and we believe that there were sufficient observations to draw major conclusion from this explorative study.

The findings are empirical which can generate new hypothesis, and the study was not designed to assess possible pathophysiological mechanisms. Furthermore, the reported sex-related differences are similar to those previously reported for the adipokine leptin [35].

### Summary and conclusions

In summary, we observed sex-related differences in MBL levels and kinetics related to sepsis survival. High levels or an increase of MBL in the acute phase of sepsis were associated with unfavourable outcome in women. We conclude that further evaluation of MBL response in acute sepsis should include a differentiated analysis with regard to gender. Further aspects of MBL response in sepsis needs to be elucidated before substitution with recombinant MBL or inhibitory therapy is considered in the future.

## Supplementary information

**Additional file 1: Figure S7.** Receiver operating characteristics, ROC analysis. ROC curve (left panel) for MBL concentrations in the acute phase (MBL2) in relation to in-hospital death for all patients (*n* = 122). In right panel sensitivity and specificity are shown for different MBL concentrations. An “optimal” cut-off point is shown in point A with a MBL concentration of 1319 ng/mL with a diagnostic accuracy of 65.6% and a sensitivity of 68.1% and a specificity of 57.1%. Area under curve is 0.60 (0.48–0.72, 95%CI), *p* = 0.044.

**Additional file 2: Figure S8.** Receiver operating characteristics, ROC analysis. ROC curve (left panel) for MBL concentration in the acute phase (MBL2) for women (*n* = 75) in relation to in-hospital death. In right panel sensitivity and specificity are shown for different MBL concentrations. An “optimal” cut-off point is shown in point A with a MBL concentration of 1319 ng/mL with a diagnostic accuracy of 73.3% and a sensitivity of 72.4% and a specificity of 76.5%. Area under curve is 0.73 (0.59–0.86, 95%CI), *p* = 0.0008.

**Additional file 3: Figure S9.** ROC curve (left panel) for MBL concentration in the acute phase (MBL2) for men (*n* = 47) in relation to in-hospital death. In right panel sensitivity and specificity are shown for different MBL concentrations. Area under curve is 0.42 (0.24–0.60, 95%CI), *p* = 0.81.

**Additional file 4: Figure S10.** ROC curve (left panel) for the difference in MBL concentration between the acute phase (MBL2) and baseline (MBL1) for women (*n* = 75) in relation to in-hospital death. In right panel sensitivity and specificity are shown. An “optimal” cut-off point is shown in point A with a MBL concentration difference of 516 ng/mL with a diagnostic accuracy of 73.3% and a sensitivity of 75.9% and a specificity of 64.7%. Area under curve is 0.74 (0.61–0.87, 95%CI), *p* = 0.0001.

**Additional file 5: Figure S11.** ROC curve (left panel) for the difference in MBL concentration between the acute phase (MBL2) and baseline (MBL1) for men (*n* = 47) in relation to in-hospital death. In right panel sensitivity and specificity are shown. Area under curve is 0.45 (0.26–0.63, 95%CI), *p* = 0.71.

**Additional file 6: Table S4.** MBL at baseline and risk for future sepsis development.

**Additional file 7: Table S5.** Comparison of MBL distribution at baseline with a cohort of Danish blood donor.

**Additional file 8: Table S6.** Comparison of MBL distribution in the acute phase with a cohort of Danish blood donor.

## Data Availability

All results and data are kept in the section of Anesthesiology and Intensive Care Medicine, Department of Surgical and Perioperative Science; Umeå University. These will be made available from the corresponding author on reasonable request.
