# Supplementary figures and images for: Levels of mannose-binding lectin (MBL) associates with sepsis-related in-hospital mortality in women
Source: J Inflamm (Lond). 2020 Aug 12;17:28. doi: 10.1186/s12950-020-00257-1 (PMC7425558; doi:10.1186/s12950-020-00257-1)

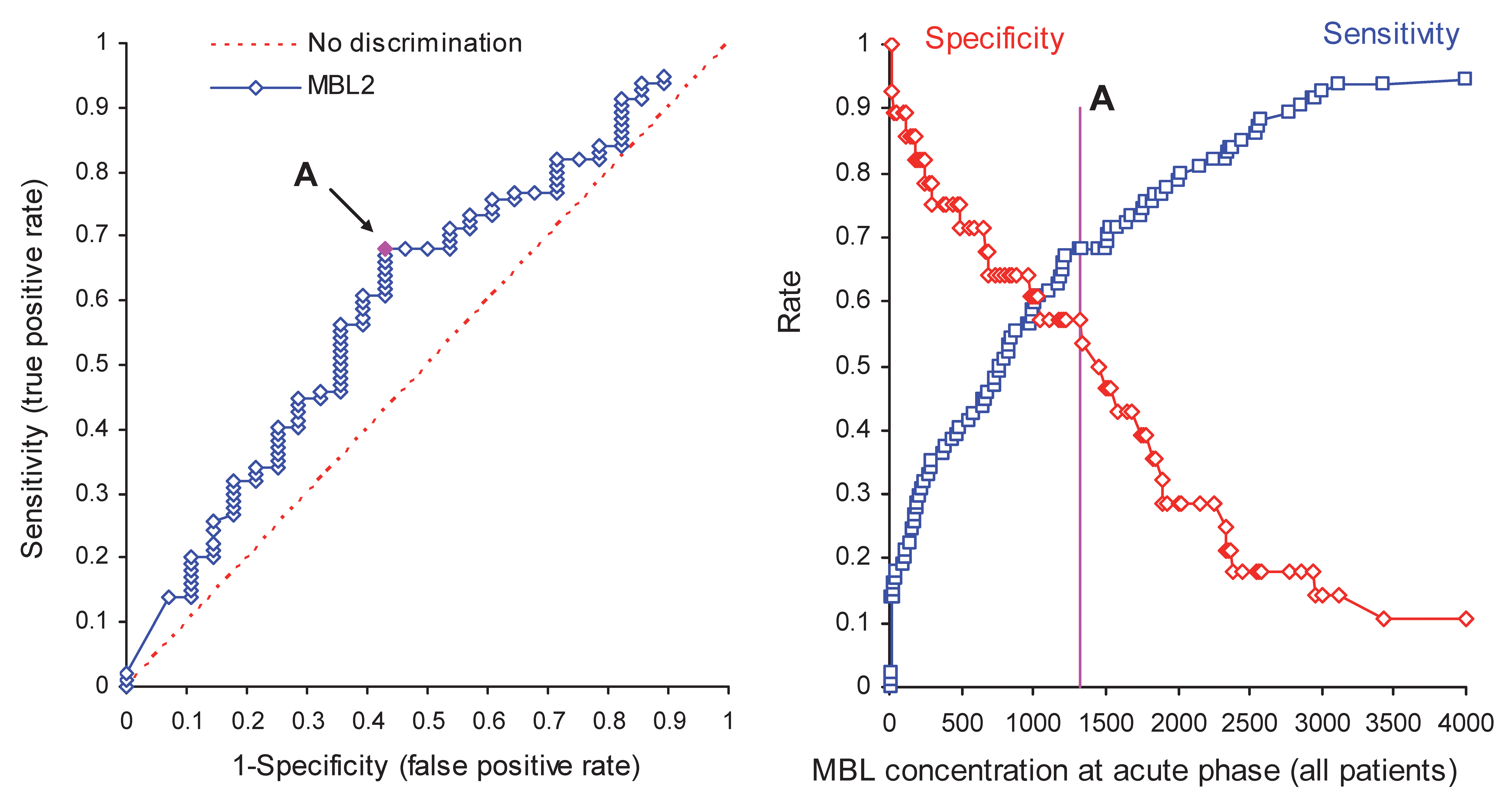

Supplement: Supplementary file 1 — Additional file 1: Figure S7. Receiver operating characteristics, ROC analysis. ROC curve (left panel) for MBL concentrations in the acute phase (MBL2) in relation to in-hospital death for all patients (n = 122). In right panel sensitivity and specificity are shown for different MBL concentrations. An “optimal” cut-off point is shown in point A with a MBL concentration of 1319 ng/mL with a diagnostic accuracy of 65.6% and a sensitivity of 68.1% and a specificity of 57.1%. Area under curve is 0.60 (0.48–0.72, 95%CI), p = 0.044. [file 12950_2020_257_MOESM1_ESM.tif]

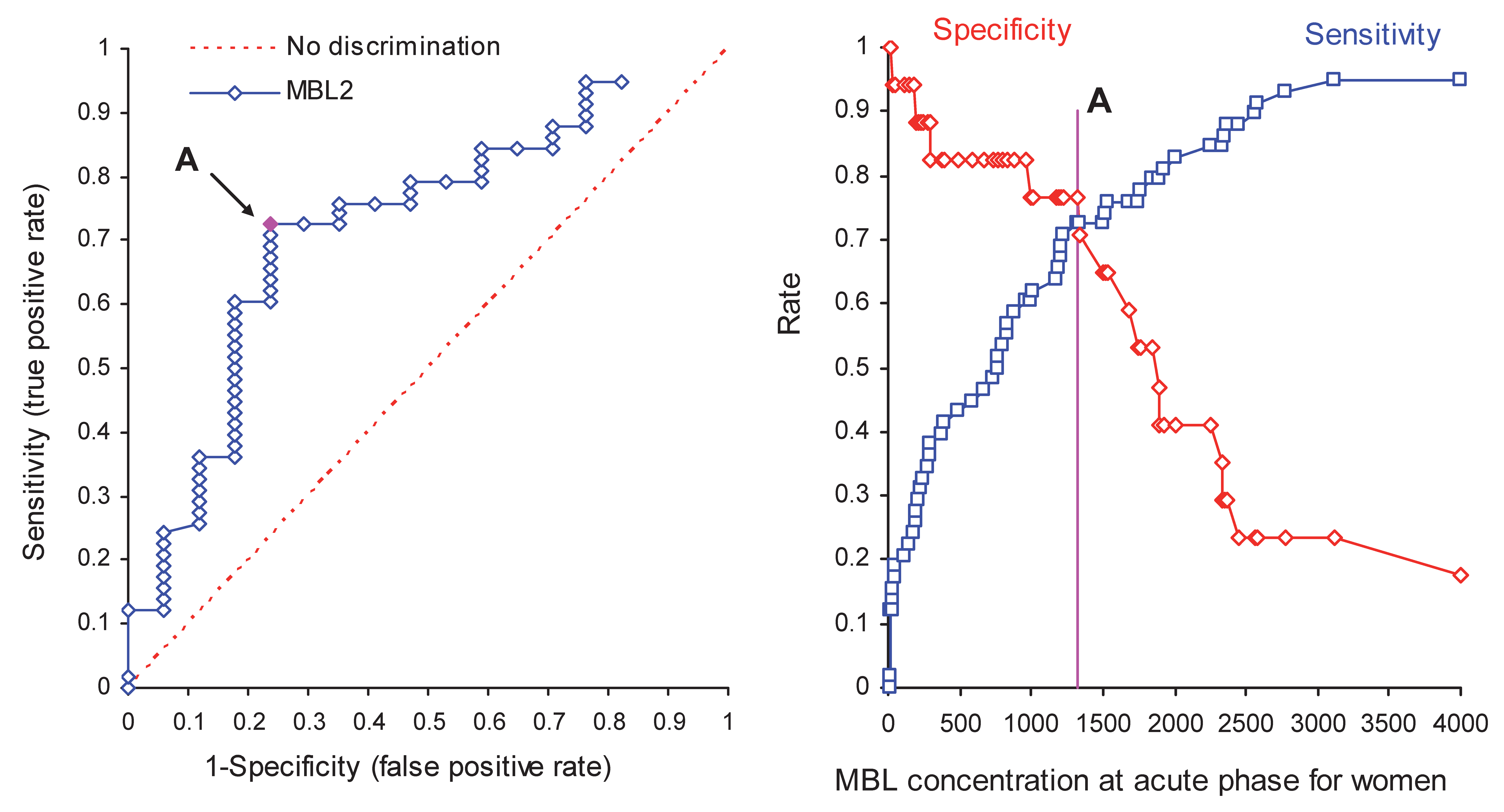

Supplement: Supplementary file 2 — Additional file 2: Figure S8. Receiver operating characteristics, ROC analysis. ROC curve (left panel) for MBL concentration in the acute phase (MBL2) for women (n = 75) in relation to in-hospital death. In right panel sensitivity and specificity are shown for different MBL concentrations. An “optimal” cut-off point is shown in point A with a MBL concentration of 1319 ng/mL with a diagnostic accuracy of 73.3% and a sensitivity of 72.4% and a specificity of 76.5%. Area under curve is 0.73 (0.59–0.86, 95%CI), p = 0.0008. [file 12950_2020_257_MOESM2_ESM.tif]

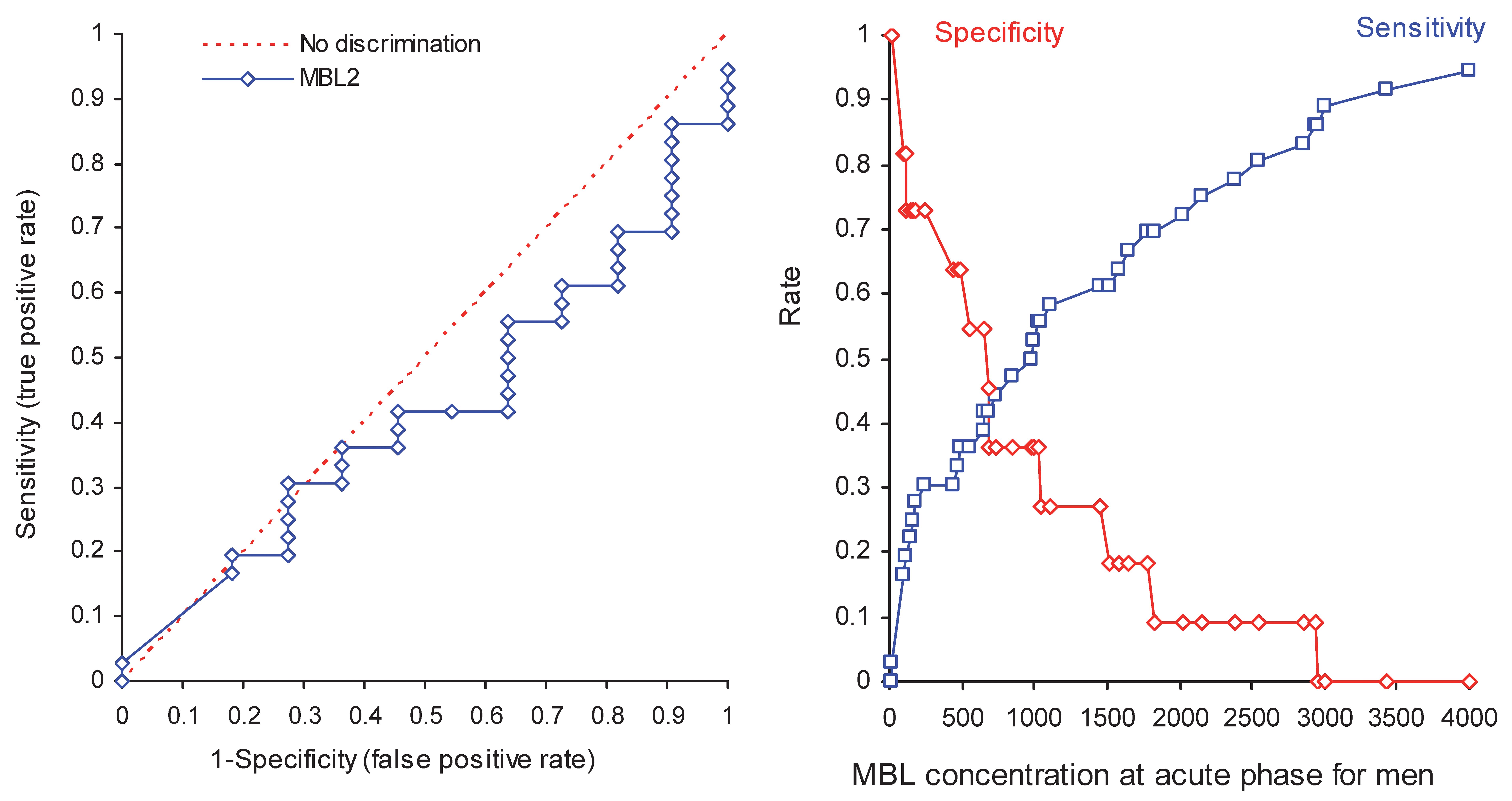

Supplement: Supplementary file 3 — Additional file 3: Figure S9. ROC curve (left panel) for MBL concentration in the acute phase (MBL2) for men (n = 47) in relation to in-hospital death. In right panel sensitivity and specificity are shown for different MBL concentrations. Area under curve is 0.42 (0.24–0.60, 95%CI), p = 0.81. [file 12950_2020_257_MOESM3_ESM.tif]

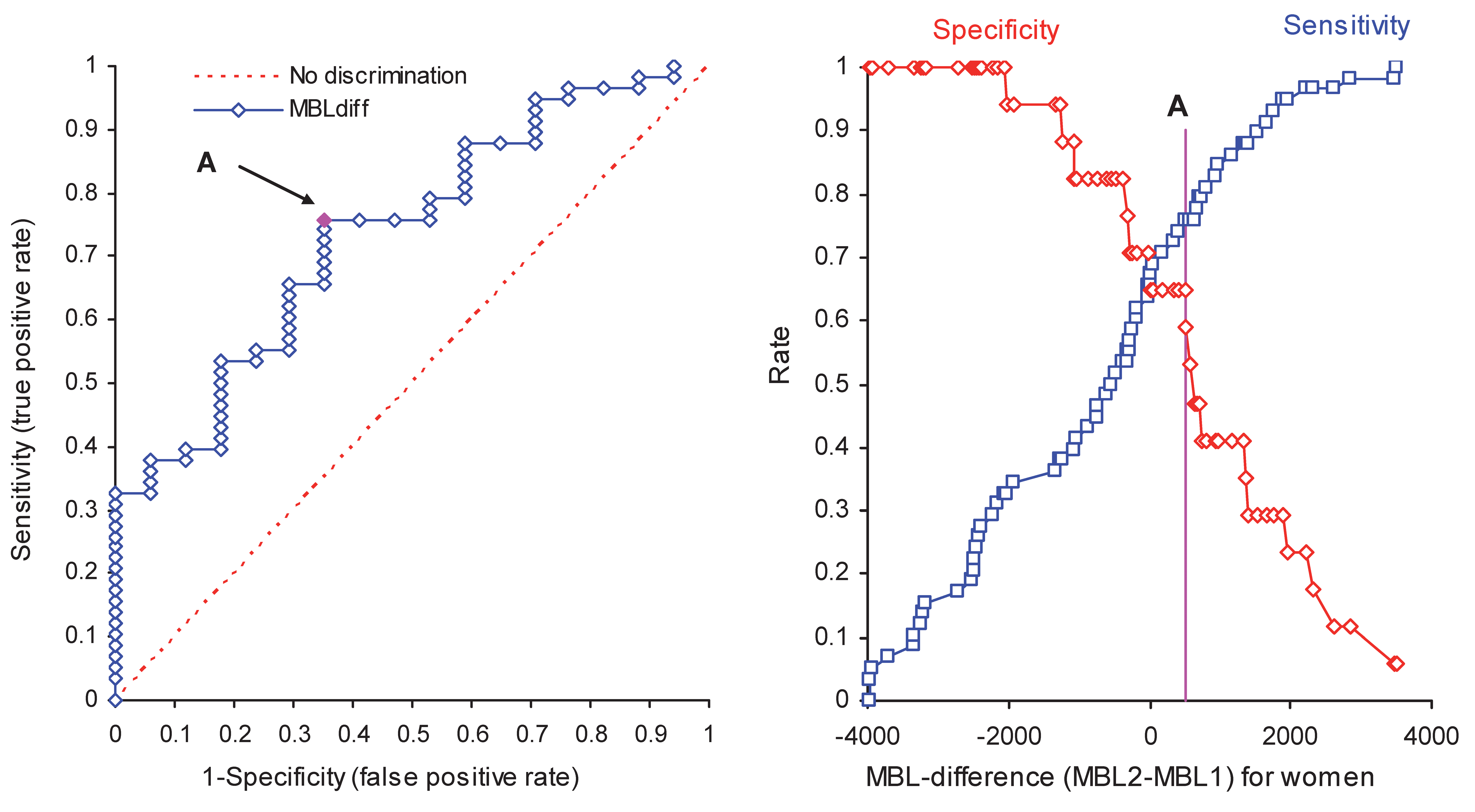

Supplement: Supplementary file 4 — Additional file 4: Figure S10. ROC curve (left panel) for the difference in MBL concentration between the acute phase (MBL2) and baseline (MBL1) for women (n = 75) in relation to in-hospital death. In right panel sensitivity and specificity are shown. An “optimal” cut-off point is shown in point A with a MBL concentration difference of 516 ng/mL with a diagnostic accuracy of 73.3% and a sensitivity of 75.9% and a specificity of 64.7%. Area under curve is 0.74 (0.61–0.87, 95%CI), p = 0.0001. [file 12950_2020_257_MOESM4_ESM.tif]

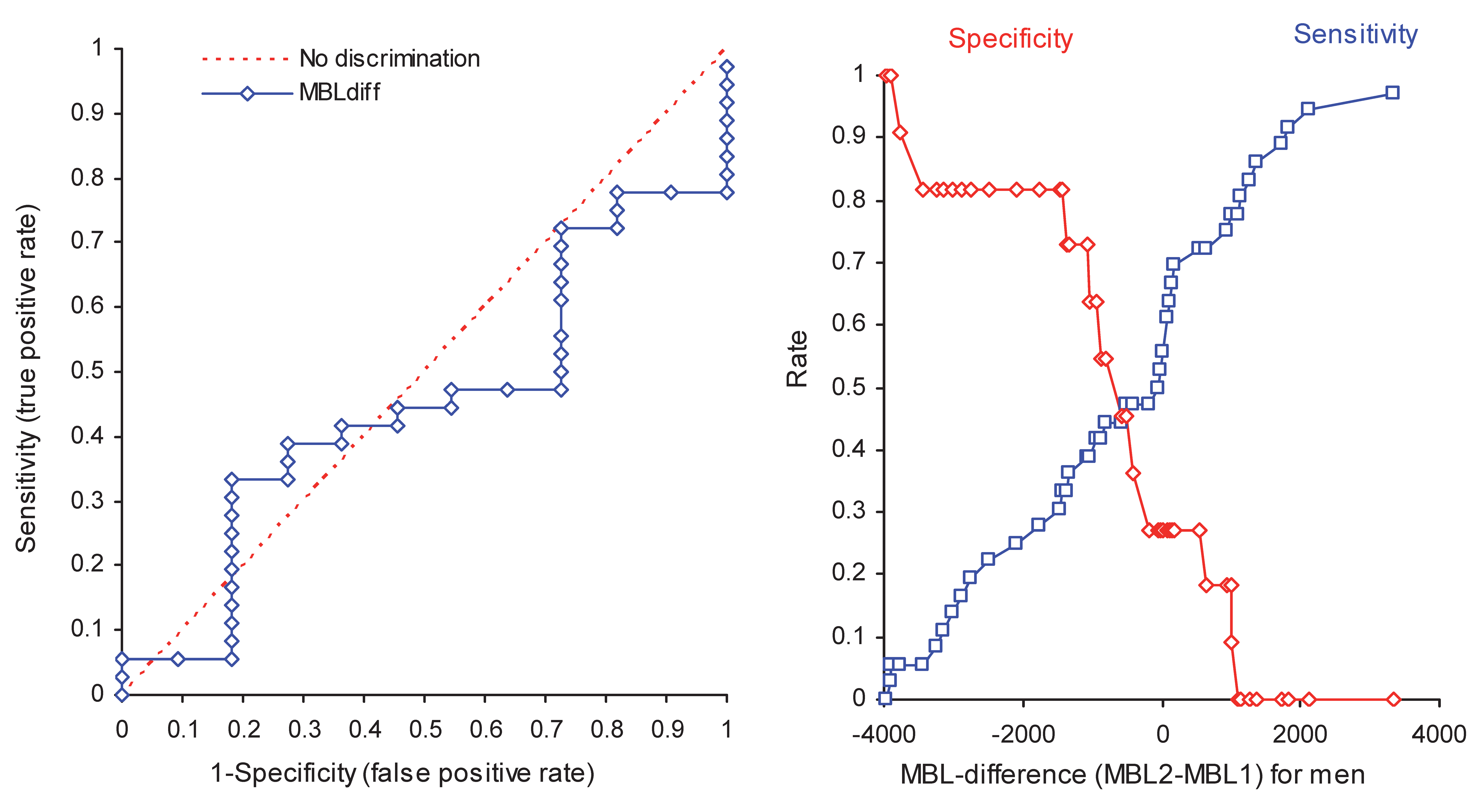

Supplement: Supplementary file 5 — Additional file 5: Figure S11. ROC curve (left panel) for the difference in MBL concentration between the acute phase (MBL2) and baseline (MBL1) for men (n = 47) in relation to in-hospital death. In right panel sensitivity and specificity are shown. Area under curve is 0.45 (0.26–0.63, 95%CI), p = 0.71. [file 12950_2020_257_MOESM5_ESM.tif]
