# Supplementary material for: Levels of mannose-binding lectin (MBL) associates with sepsis-related in-hospital mortality in women
Source: J Inflamm (Lond). 2020 Aug 12;17:28. doi: 10.1186/s12950-020-00257-1 (PMC7425558; doi:10.1186/s12950-020-00257-1)
Supplement: Supplementary file 6 — Additional file 6: Table S4. MBL at baseline and risk for future sepsis development. [file 12950_2020_257_MOESM6_ESM.doc]

Supplementary Table 4. MBL at baseline and risk for future sepsis development.

|  |  |  | **All** |  |  |  |  | **Men** |  |  |  |  | **Women** |  |  |  |
| --- | --- | --- | --- | --- | --- | --- | --- | --- | --- | --- | --- | --- | --- | --- | --- | --- |
|  | MBL ng/mL |  | Referents | Cases | OR | 95% CI |  | Referents | Cases | OR | 95% CI |  | Referents | Cases | OR | 95% CI |
|  |  |  |  |  |  |  |  |  |  |  |  |  |  |  |  |  |
| **All** | <50 |  | 28 | 21 | 1.00 |  |  | 8 | 7 | 1.00 |  |  | 20 | 14 | 1.00 |  |
|  | >50 |  | 268 | 127 | 0.65 | 0.36-1.17 |  | 106 | 50 | 0.57 | 0.21-1.58 |  | 162 | 77 | 0.69 | 0.34-1.42 |
|  |  |  |  |  |  |  |  |  |  |  |  |  |  |  |  |  |
|  | <100 |  | 37 | 23 | 1.00 |  |  | 14 | 9 | 1.00 |  |  | 23 | 14 | 1.00 |  |
|  | 100-1000 |  | 87 | 32 | 0.61 | 0.32-1.15 |  | 26 | 11 | 0.66 | 0.22-1.97 |  | 61 | 21 | 0.59 | 0.27-1.30 |
|  | >1000 |  | 172 | 93 | 0.89 | 0.50-1.58 |  | 74 | 37 | 0.78 | 0.31-1.96 |  | 98 | 56 | 0.99 | 0.48-2.05 |
|  |  |  |  |  |  |  |  |  |  |  |  |  |  |  |  |  |
| **Severe sepsis** | <50 |  | 16 | 15 | 1.00 |  |  | 5 | 6 | 1.00 |  |  | 11 | 9 | 1.00 |  |
|  | >50 |  | 182 | 84 | 0.51 | 0.24-1.06 |  | 79 | 36 | 0.42 | 0.13-1.36 |  | 103 | 48 | 0.57 | 0.22-1.47 |
|  |  |  |  |  |  |  |  |  |  |  |  |  |  |  |  |  |
|  | <100 |  | 22 | 17 | 1.00 |  |  | 9 | 8 | 1.00 |  |  | 13 | 9 | 1.00 | 0 |
|  | 100-1000 |  | 49 | 19 | 0.51 | 0.22-1.15 |  | 17 | 8 | 0.50 | 0.13-1.88 |  | 32 | 11 | 0.52 | 0.18-1.48 |
|  | >1000 |  | 127 | 63 | 0.64 | 0.32-1.31 |  | 58 | 26 | 0.48 | 0.16-1.46 |  | 69 | 37 | 0.80 | 0.32-2.03 |
|  |  |  |  |  |  |  |  |  |  |  |  |  |  |  |  |  |
| **Septic shock** | <50 |  | 12 | 6 | 1.00 | 0.36-2.74 |  | 3 | 1 | 1.00 |  |  | 9 | 5 | 1.00 | 0.36-3.49 |
|  | >50 |  | 86 | 43 | 1.00 |  |  | 27 | 14 | 1.50 | 0.16-14.42 |  | 59 | 29 | 0.89 | 0.29-2.78 |
|  |  |  |  |  |  |  |  |  |  |  |  |  |  |  |  |  |
|  | <100 |  | 22 | 17 | 1.00 |  |  | 5 | 1 | 1.00 |  |  | 10 | 5 | 1.00 |  |
|  | 100-1000 |  | 49 | 19 | 0.87 | 0.30-2.53 |  | 9 | 3 | 1.59 | 0.14-17.52 |  | 29 | 10 | 0.72 | 0.22-2.43 |
|  | >1000 |  | 127 | 63 | 1.70 | 0.61-4.70 |  | 16 | 11 | 3.01 | 0.34-26.51 |  | 29 | 19 | 1.39 | 0.42-4.60 |
|  |  |  |  |  |  |  |  |  |  |  |  |  |  |  |  |  |
| **Hospital dead** | <50 |  | 9 | 6 | 0.72 | 0.23-2.20 |  | 2 | 1 | 1.00 | 0.09-11.03 |  | 7 | 5 | 0.65 | 0.18-2.33 |
|  | >50 |  | 55 | 26 | 1.00 |  |  | 24 | 12 | 1.00 |  |  | 31 | 14 | 1.00 |  |
|  |  |  |  |  |  |  |  |  |  |  |  |  |  |  |  |  |
|  | <100 |  | 13 | 6 | 1.00 |  |  | 6 | 1 | 1.00 |  |  | 7 | 5 | 1.00 |  |
|  | 100-1000 |  | 17 | 10 | 1.24 | 0.37-4.15 |  | 7 | 6 | 5.06 | 0.47-54.80 |  | 10 | 4 | 0.60 | 0.13-2.78 |
|  | >1000 |  | 34 | 16 | 0.99 | 0.30-3.29 |  | 13 | 6 | 3.13 | 0.28-34.53 |  | 21 | 10 | 0.68 | 0.16-2.94 |

Conditional logistic regression analysis. Values shown are Odds ratios (OR) with 95% CI for MBL at baseline and risk for a future sepsis event.

MBL were first categorized into a dicotomuos variable with a cut-off at 50 ng/ml: <50 ng/ml (low) or >50 ng/ml (high).

MBL was then categorised as low, medium and high with cut offs as follows: <100 ng/ml (low), 100-1000 ng/ml (medium) or >1000 ng/ml (high).

The ORs represent the risk associated with the medium and high levels of MBL, respectively, versus the low levels (reference with OR 1.00). Univariate analyses only
