# Supplementary material for: Levels of mannose-binding lectin (MBL) associates with sepsis-related in-hospital mortality in women
Source: J Inflamm (Lond). 2020 Aug 12;17:28. doi: 10.1186/s12950-020-00257-1 (PMC7425558; doi:10.1186/s12950-020-00257-1)
Supplement: Supplementary file 7 — Additional file 7: Table S5. Comparison of MBL distribution at baseline with a cohort of Danish blood donor. [file 12950_2020_257_MOESM7_ESM.doc]

Supplementary Table 5. Comparison of MBL distribution at baseline with a cohort of Danish blood donors**.**

|  | Low | Medium | High | All | Chi2 | *P* |
| --- | --- | --- | --- | --- | --- | --- |
| MBL (ng/mL) | <100 | 100-1000 | >1000 |  |  |  |
|  |  |  |  |  |  |  |
| Danish study n (%) | 18 (16.7) | 30 (27.8) | 60 (55.5) | 108 (100) |  |  |
|  |  |  |  |  |  |  |
| Our study |  |  |  |  |  |  |
| Referents n (%) | 37 (12.5) | 87 (29.4) | 172 (58.1) | 296 (100) | 1.17 | 0.56 |
| Cases n (%) | 23 (15.5) | 32 (21.6) | 93 (62.8) | 148 (100) | 1.58 | 0.45 |
| All n (%) | 60 (13.5) | 119 (26.8) | 265 (59.7) | 444 (100) | 0.89 | 0.64 |
|  |  |  |  |  |  |  |
| Referents (men) n (%) | 14 (12.3) | 26 (22.8) | 74 (64.9) | 114 (100) | 2.09 | 0.35 |
| Cases (men) n (%) | 9 (15.8) | 11 (19.3) | 37 (64.9) | 57 (100) | 1.65 | 0.44 |
|  |  |  |  |  |  |  |
| Referents (women) n (%) | 23 (12.6) | 61 (33.5) | 98 (53.8) | 182 (100) | 1.53 | 0.47 |
| Cases (women) n (%) | 14 (15.4) | 21 (23.1) | 56 (61.5) | 91 (100) | 0.78 | 0.68 |

Comparisons of 108 healthy Danish blood donors with our study cohort at baseline (health survey), 148 cases and 296 referents. Data provided by the manufacturer of the ELISA kit used for MBL analysis. The MBL distribution expressed as low, medium and high, according to the manufacturer.. The P-value represents comparison with the Danish blood donors for each row separately, ie Danes vs referents, Danes vs cases, Danes vs all, Danes vs male referents and cases separately, Danes vs women referents and cases separately. Chi2-test were used to compare groups
