# Supplementary material for: Levels of mannose-binding lectin (MBL) associates with sepsis-related in-hospital mortality in women
Source: J Inflamm (Lond). 2020 Aug 12;17:28. doi: 10.1186/s12950-020-00257-1 (PMC7425558; doi:10.1186/s12950-020-00257-1)
Supplement: Supplementary file 8 — Additional file 8: Table S6. Comparison of MBL distribution in the acute phase with a cohort of Danish blood donor. [file 12950_2020_257_MOESM8_ESM.doc]

Supplementary Table 6**.** Comparison of MBL distribution in the acute phase with a cohort of Danish blood donors.

|  | Low | Medium | High | All | Chi2 | *P* |
| --- | --- | --- | --- | --- | --- | --- |
| MBL (ng/mL) | <100 | 100-1000 | >1000 |  |  |  |
|  |  |  |  |  |  |  |
| Danish study n (%) | 18(16.7) | 30(27.8) | 60(55.5) | 108 (100) |  |  |
|  |  |  |  |  |  |  |
| Our study |  |  |  |  |  |  |
| All n (%) | 22 (17.7) | 44 (35.5) | 56 (45.2) | 122 (100) | 2.34 | 0.31 |
| Men n (%) | 9 (18.8) | 18 (37.5) | 20 (41.7) | 47 (100) | 2.36 | 0.31 |
| Women n (%) | 13 (17.1) | 26 (34.2) | 36 (47.4) | 75 (100) | 1.18 | 0.55 |

Comparisons of 108 healthy Danish blood donors with our study cohort in the acute phase (sepsis event), 122 cases. MBL distribution expressed as low, medium and high, according to the manufacturer of the ELISA kit used to analyze MBL. The P-value represents comparison with the Danish blood donors for each row separately, ie Danes vs all, Danes vs men, Danes vs women. Chi2-test were used to compare groups
